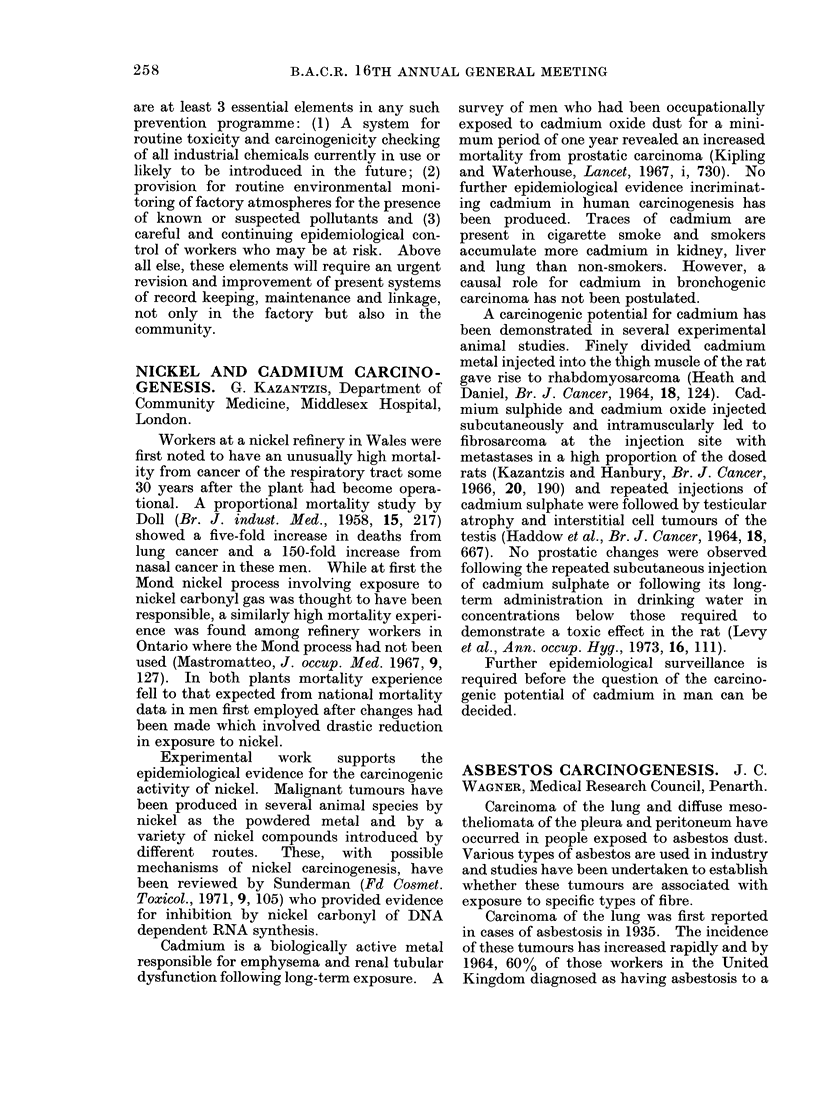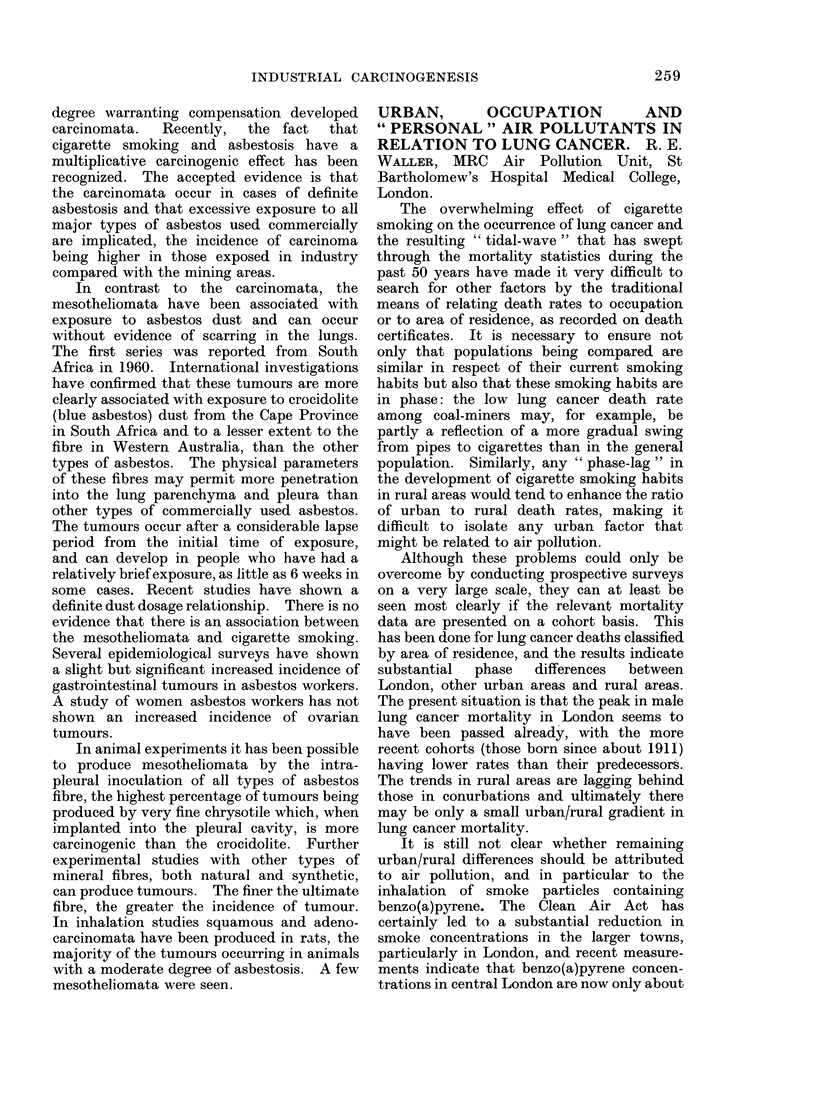# Proceedings: Asbestos carcinogenesis.

**DOI:** 10.1038/bjc.1975.206

**Published:** 1975-08

**Authors:** J. C. Wagner


**ASBESTOS CARCINOGENESIS.** J. C. WAGNER, Medical Research Council, Penarth.

Carcinoma of the lung and diffuse mesotheliomata of the pleura and peritoneum have occurred in people exposed to asbestos dust. Various types of asbestos are used in industry and studies have been undertaken to establish whether these tumours are associated with exposure to specific types of fibre.

Carcinoma of the lung was first reported in cases of asbestosis in 1935. The incidence of these tumours has increased rapidly and by 1964, 60% of those workers in the United Kingdom diagnosed as having asbestosis to a degree warranting compensation developed carcinomata. Recently, the fact that cigarette smoking and asbestosis have a multiplicative carcinogenic effect has been recognized. The accepted evidence is that the carcinomata occur in cases of definite asbestosis and that excessive exposure to all major types of asbestos used commercially are implicated, the incidence of carcinoma being higher in those exposed in industry compared with the mining areas.

In contrast to the carcinomata, the mesotheliomata have been associated with exposure to asbestos dust and can occur without evidence of scarring in the lungs. The first series was reported from South Africa in 1960. International investigations have confirmed that these tumours are more clearly associated with exposure to crocidolite (blue asbestos) dust from the Cape Province in South Africa and to a lesser extent to the fibre in Western Australia, than the other types of asbestos. The physical parameters of these fibres may permit more penetration into the lung parenchyma and pleura than other types of commercially used asbestos. The tumours occur after a considerable lapse period from the initial time of exposure, and can develop in people who have had a relatively brief exposure, as little as 6 weeks in some cases. Recent studies have shown a definite dust dosage relationship. There is no evidence that there is an association between the mesotheliomata and cigarette smoking. Several epidemiological surveys have shown a slight but significant increased incidence of gastrointestinal tumours in asbestos workers. A study of women asbestos workers has not shown an increased incidence of ovarian tumours.

In animal experiments it has been possible to produce mesotheliomata by the intrapleural inoculation of all types of asbestos fibre, the highest percentage of tumours being produced by very fine chrysotile which, when implanted into the pleural cavity, is more carcinogenic than the crocidolite. Further experimental studies with other types of mineral fibres, both natural and synthetic, can produce tumours. The finer the ultimate fibre, the greater the incidence of tumour. In inhalation studies squamous and adenocarcinomata have been produced in rats, the majority of the tumours occurring in animals with a moderate degree of asbestosis. A few mesotheliomata were seen.